# Characterisation of the surface growth of *Mucor circinelloides* in cheese agar media using predictive mathematical models

**DOI:** 10.1016/j.heliyon.2024.e30812

**Published:** 2024-05-07

**Authors:** Martina Koňuchová, Agáta Boháčiková, Ľubomír Valík

**Affiliations:** Institute of Food Sciences and Nutrition, Faculty of Chemical and Food Technology, Slovak University of Technology Bratislava, Radlinského 9, SK-812 37, Bratislava, Slovakia

## Abstract

The main objective of this work was to characterise the mycelial growth of *Mucor circinelloides*, one of the fungal contaminants that appear frequently in the artisan cheese production environment. The study uses primary Baranyi and Huang models to compare their parameters and predict *M. circinelloides* on cheese-based medium (CBA) under diverse environmental conditions (temperature range from 6 to 37 °C and 0 and 1 % NaCl concentration). However, the Baranyi model consistently estimated longer lag phases and higher surface growth rates (*sgr*) than the Huang model; both models showed adequate best-fit performance (exactly with the mean coefficient of determination *R*^*2*^ = (0.993 ± 0.020 × 10^−1^). The groups of primary growth parameters were analysed against temperature using the cardinal model (CM) with the following main outputs. The optimal surface growth rates (*sgr*_opt_) on CBA were 6.8 and 6.5 mm/d calculated with the Baranyi and Huang models, respectively. They were reduced by approximately 46 % on the surface of the agar medium when 1 % NaCl was added. *T*_opt_ was estimated in a very narrow range of 32.1–32.5 °C from both primary *sgr* data sets (0 % and 1 % NaCl). Similarly, *T*_max_ values of 37.2 °C and 37.3 °C were estimated for the Baranyi and Huang models, respectively; however, they decreased at 2 °C in CBA with 1 % NaCl (*T*_max_ = 35.1 °C). The application of CM for sgr provided an estimation of the parameter *T*_min_ with negative values that are considered only as a theoretical output. The results provide insight into the modelling and prediction of fungi growth as a function of time and salt concentration, including the times to detect visible mycelial growth of *Mucor circinelloides*. The mere quantification of this phenomenon can be useful for practice. Adjusting the frequency of the cheese surface washing step with a salt solution at the early stage of ripening properly can prevent the growth of not only fast fungal growers.

## 1 Introduction

The growth of fungi in cheese represents quality and safety issues and leads to food loss and waste with significant economic impacts. Physiological characteristics contribute to the ability of fungi to grow at low refrigeration temperatures, higher salt concentration (low water activity (*a*_*w*_) values), lower pH, lower oxygen tension atmospheres, and nutrient limitations [[1], [2], [3], [4]]. The frequently reported dairy-relevant spoilage fungi are species belonging to the phylum *Ascomycota* and *Zygomycota* (*Mucoromycota)*. *Mucor* spp. are significant spoilage agents of concern in fresh or short-ripening cheese matrices [2,3,[5], [6], [7], [8], [9]]. Some of the *Mucor* species can grow under anaerobic conditions but this hardly is the case with cheese packed in a vacuum or modified atmosphere. Furthermore, they are well adapted to the dairy environment, lower temperatures and pH [[10], [11], [12], [13], [14], [15]]. In the context of cheese making, *Mucor circinelloides* van Tieghem is recognised as a source of spoilage of produce. This fungus is a dimorphic organism that can switch between the yeast-like and hyphal phase, depending on the environmental conditions [14,[16], [17], [18], [19]]. Several species of *Mucor* are known to cause diseases. *M. circinelloides* was recorded as an etiologic agent of mucormycosis in immunosuppressed patients after ingestion of contaminated yoghurt [[20], [21], [22]].

Predictive microbiology is considered a highly relevant concept for the quantitative description and prediction of microbial behaviour in foods. It also belongs to the most important decisive tools used in microbiological food safety and quality assurance. Among several approaches such as measurements of hyphal length, colony diameter, heat production rate, colony biomass, and amount of chitin or ergosterol that have been developed to quantify the growth of filamentous fungi on solid substrates, the most common and simplest is the direct measurement of colony diameter over time [23,24].

The main objective of this work was to characterise the mycelial growth of *M. circinelloides*, one of the fungal contaminants that frequently appear in the artisan cheese production environment and the surface of the cheese. In the context of predictive mycology, the model proposed by Baranyi et al. [25] has originally been developed to describe bacterial growth; however, many authors confirmed its accurate prediction capability for fungal growth [[26], [27], [28], [29], [30], [31], [32], [33], [34], [35], [36], [37], [38]]. Following a detailed mechanistic approach, Huang developed another primary growth model [39,40], which is grounded in the fundamental growth phenomenon of microorganisms and clearly defines the duration of the lag phase and the exponential growth rate [41]. Despite being an alternative to the model proposed by Baranyi and Roberts [42,43], Huang's model has been less implemented in fungal growth quantification. To the best of the authors' knowledge, only a few comparisons between the Baranyi and Huang models have been made when modelling fungal growth [44]. Therefore, in addition to providing data on the mycelial growth of *M*. *circinelloides*, this study aims to apply the Huang and Baranyi models to estimate the kinetic response of *M. circinelloides* in CBA under different environmental conditions (temperature and salt content). The other objectives were to compare the performance of the primary models and also to estimate how it will be reflected in secondary modelling. Moreover, within validation, the present study quantifies the growth of *M. circinelloides* with dynamic changes in storage temperature.

## 2 Material and methods

### 2.1 Fungal isolate and culture conditions

The fungal isolate *M*. *circinelloides* 1L was selected for its significance in fresh or short-ripened cheese products and the dairy environment. This isolate was obtained from the culture collection of the Institute of Food Science and Nutrition (Slovak University of Technology in Bratislava, Slovakia) and was originally isolated from the traditional Slovak cheese "Bryndza" [45]. The confirmation of strain 1L is based on the morphological and physiological characteristics following Pitt and Hocking [6], Botha and Botes [46], and Samson et al. [47]. The molecular identification was performed at the Institute of Molecular Biology of the Slovak Academy of Sciences and was carried out according to Pangallo et al. [48].

The stock culture was regularly propagated and maintained refrigerated (5 ± 0.5 °C) on SAB slants (Sabouraud Dextrose agar, Biolife Italiana Srl, Milan, Italy) slants. For long-term storage, the culture was kept frozen at −70 °C in tubes containing yeast malt broth (Sigma-Aldrich, St. Louis, MO, USA) supplemented with 20 % glycerol.

### 2.2 Inoculum preparation

The inoculum was prepared from a 5-day culture grown on the top layer of a perpendicular SAB agar tube at 25 ± 0.5 °C to obtain a heavily sporulating culture. A suspension was then prepared by washing the culture with sterile saline solution (8.5 g L^−1^ NaCl, 0.1 g L^−1^ of peptone) containing 0.01 % (v/v) of the Tween® 80 wetting agent (Merck KGaA, Darmstadt, Germany). Immediately after preparation, the suspension of the fungal strain was adjusted to 10^3^ spores/mL and used for inoculation.

### 2.3 Experimental design and growth studies

All experiments were performed on plates with cheese-based agar (CBA) [49,50]; with the following composition: casamino acids (15 gL^−1^) (Merck KgaG, Darmstadt, Germany), sodium lactate (38 mL L^−1^) (Merck KgaG, Darmstadt, Germany), yeast extract (1 g L^−1^) (Biolife Italiana, Milano, Italy), CaCl_2_ (0.1 g L^−1^) (Centralchem, Bratislava, Slovakia), MgSO_4_ (0.5 g L^−1^) (Centralchem, Bratislava, Slovakia), KH_2_PO_4_ (6.8 g L^−1^) (Centralchem, Bratislava, Slovakia), NaCl (Centralchem, Bratislava, Slovakia), lactose (28 g L^−1^) (Merck KgaG, Darmstadt, Germany) and l-methionine (6 g L^−1^) (Merck KgaG, Darmstadt, Germany) and agar-agar (Merck KgaG, Darmstadt, Germany). After sterilisation, 50 mL of medium was poured into sterile Petri dishes (diameter 130 mm). Then 2 μL of *M. circinelloides* spore suspensions were used to inoculate the centre of each CBA plate. In this study, the initial diameter (*d*_0_) of the inoculated spore suspension was established at 2.5 mm. For all experiments, time zero was defined as the time the suspension was applied to the surface of the agar plate. After inoculation, three parallel plates were sealed in polyethylene bags to prevent water loss.

The diameters of developing colonies were measured at appropriate time intervals, using a Vernier calliper (150 mm × 0.02 mm; Sinochem Jiangsu, Nanjing, China) in two directions at right angles to each other, without opening the dishes. The final diameter of the colonies (expressed in mm) was calculated as an arithmetic mean. Measurements were taken from the early stages of growth to capture the lag phase.

Two sets of experimental conditions were implemented, one under constant temperatures and the other non-isothermal, both in two different *a*_*w*_ scenarios: 0.974 ± 0.002 (unmodified growth medium) and 0.963 ± 0.003 (1 % NaCl, w/v). The *a*_*w*_ measurements were performed with LabMaster-aw (Novasina, Lachen, Switzerland).

For isothermal incubation, plates were stored at temperatures of 6, 8, 12, 15, 18, 21, 25, 30, 33, 35, and 37 ± 0.5 °C in incubators (Pol-Eko Aparatura, Wodzisław Śląski, Poland) under static aerobic conditions. The following temperature levels were selected to cover the growth region of the species to the maximum extent possible.

The validation study was carried out under dynamic temperature regimes (25 °C/48 h, 18 °C/48 h, 15 °C/120 h, 10 °C/120 h), which simulates the time and temperature conditions during the ripening of lump cheese, a raw material for Bryndza cheese [51]. The non-isothermal temperature profile may also cover the fluctuating conditions that may occur during other cheese manufacturing at their early ripening phase. The high-precision programmable incubator (model ES-20/80C; Biosan SIA, Riga, Latvia) was used to ensure the above time/temperature conditions.

### 2.4 Growth data modelling

#### 2.4.1 Primary modelling

Two primary models, Baranyi and Roberts [42,43] and Huang [39,40] were used to fit the experimental data from each growth curve. The sigmoidal Baranyi function was analysed using the Excel-based tool (Microsoft Excel, Redmond, WD, USA) ‘DMFit’ version 3.5 (ComBase managed by the United States Department of Agriculture-Agricultural Research Service, Washington, USA; and the University of Tasmania Food Safety Centre, Hobart, Australia). The reparametrized Baranyi model for fungal growth is described by the following equation.

(1) $$d\left(t\right)=s{gr}_{\max}∙A\left(t\right)-\log\left\{1+\frac{\exp\left[{sgr}_{\max}∙A\left(t\right)\right]-1}{\exp\left[d_{\max}-d_{0}\right]}\right\}$$

(2) $$A\left(t\right)=t+\frac{1}{{sgr}_{\max}}∙\log\;\left[\exp\;\left(-{sgr}_{\max}∙t\right)+\exp\;\left(-{sgr}_{\max}∙\lambda \right)-\exp\;\left(-{sgr}_{\max}∙t-s{gr}_{\max}∙\lambda \right)\right]$$

where d(t) is the colony diameter (mm) in actual time, ${sgr}_{\;\max}$ is the maximum surface growth rate (mm.h^−1^), λ (h) is the lag phase duration, $d_{\max}$ is the final colony diameter and $d_{0}$ is the diameter in time zero. This model can describe growth curves either with or without the lag phase and with or without the stationary phase. This curvature is controlled by two parameters, namely, upper asymptote (*n*) and lower asymptote (*m*). The *m* curvature and *n* curvature parameters were set to 10, by default. The equation in identical form was applied also by Zardetto et al. [52].

Huang's model was solved numerically as a differential equation using the analysis tool Solver incorporated into Microsoft Excel 365. The following reparametrized equation was used for fungal growth:

(3) $$d\left(t\right)=d_{0}+d_{\max}-\ln\left\{e^{d_{0}}\left[e^{d_{\max}}-e^{d_{0}}\right]e^{-s{gr}_{\max}B\left(t\right)}\right\}$$

(4) $$B\left(t\right)=t+\frac{1}{\alpha }\ln\frac{1+e^{-\alpha \left(t-\lambda \right)}}{1+e^{\alpha \lambda }}$$

where B(t) is the transition function, t is time, and α is the lag phase transition coefficient. The parameter α was set at 4 as suggested by the authors [40].

#### 2.4.2 Secondary modelling

The estimates of *sgr*_max_ and *λ* were modelled with the secondary cardinal model (CM; Eqs. (5), (6)), respectively) by Rosso et al. [53] to describe the effects of temperature on fungal growth at the *a*_*w*_ values of 0.974 and 0.963 representing the *a*_*w*_ value of CBA and the medium with 1 % addition of NaCl (w/v), respectively.

(5) $$sgr={sgr}_{opt}∙CM\left(T\right)$$

(6) $$\ln\frac{1}{\lambda }=\ln\frac{1}{\lambda_{opt}}∙CM\left(T\right)$$

where

(7) $$CM\left(T\right)=\left\{\frac{\left(T-T_{\max}\right){\left(T-T_{\min}\right)}^{2}}{\left(T_{opt}-T_{\min}\right)∙\left\{\left(T_{opt}-T_{\min}\right)∙\left({T-T}_{opt}\right)-\left(T_{opt}-T_{\max}\right)∙\left[T_{opt}+T_{\min}-2T\right]\right\}}\right\}$$

This secondary model includes four parameters with direct biological meaning, such as direct *sgr*_*max*_, *λ*, *T*_min_ (theoretical minimum temperature), *T*_opt_ (optimal temperature) and *T*_max_ (maximum temperature above which fungal growth is not likely). The parameters of the model, including their errors, were estimated with non-linear regression tools incorporated in Statistica vs. 10 (Tibco, Santa Clara, USA).

#### 2.4.3 Statistical analysis and model validation

Triplicated data obtained from primary modelling were treated with analysis of variance (ANOVA) to compare whether the prediction capability of the Baranyi and Huang models for the growth of *M. circinelloides* was significant. A statistical analysis with the least significant difference of 95 % was performed using Microsoft Excel 365.

To evaluate the accuracy of the response model fitting and predictions, the evaluation criteria of the coefficient of determination (*R*^*2*^) and the root mean square error (*RMSE*) evaluation criteria were used:

(8) $$R^{2}=1-\frac{SSE}{SST}=\frac{\sum_{i=1}^{n}{\left(y_{i}^{\exp}-y_{i}^{cal}\right)}^{2}}{\sum_{i=1}^{n}{\left(y_{i}^{\exp}-{\bar{y}}_{i}^{cal}\right)}^{2}}$$

(9) $$RMSE=\sqrt{\frac{SSE}{n-p}}$$

where $y_{i}^{\exp}$, $y_{i}^{cal}$ and ${\bar{y}}_{i}^{cal}$ are the experimental (observed), calculated (predicted) and average *sgr* or *λ* data, respectively; *n* is the number of experimental observations; and *p* is the number of model parameters [54,55].

The bias and accuracy factors were calculated using the following equations [56]:

(10) $$B_{f}=e^{\frac{\sum_{i=1}^{n}{(\ln\;y}_{i}^{cal}-\ln\;y_{i}^{\exp}\;)}{n}}$$

(11) $$A_{f}=e^{\sqrt{\frac{\sum_{i=1}^{n}{\left({\ln\;y}_{i}^{cal}-\ln\;y_{i}^{\exp}\right)}^{2}}{n}}}$$

Independent growth experiments were carried out in the following dynamic *combinations of t/T:* 2 d/25 °C, 2 d/18 °C, 5 d/15 °C and 5 d/10 °C) to validate the secondary CM estimated for *sgr* and *λ*. The predicted diameters of *M. circinelloides* colonies for validation experiments were calculated as the sum of the diameters calculated in each t/T combination in which the general primary growth model with only lag and exponential phase was used: $d_{cal}=d_{0}+{sgr\left(\text{CM}\right)}_{cal}∙\left(t-\lambda \right)$. Eqs. (5), (6)) were used to calculate *sgr*_cal_ and *lag*(CM)_cal_, respectively.

## 3 Results and discussion

### 3.1 Primary modelling of *M. circinelloides* mycelial growth

**Fig. 1:**
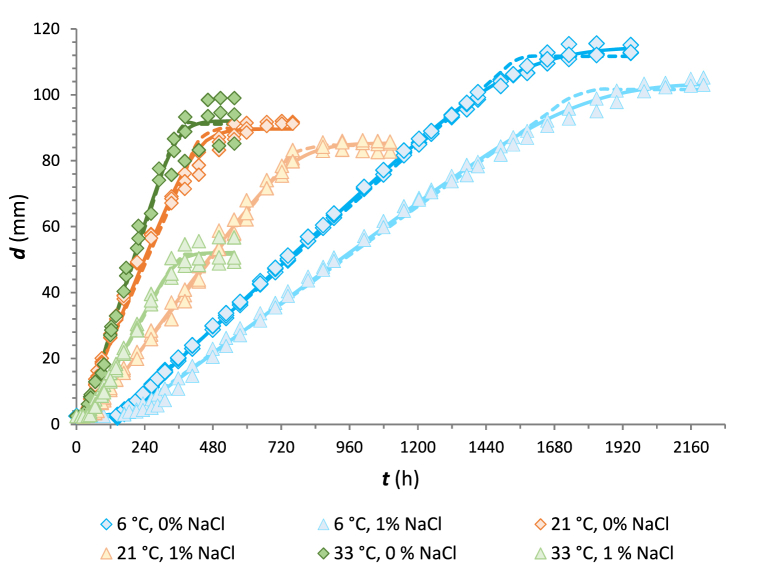
Growth curves of *M. circinelloides* 1L on CBA at 6, 21 and 33 °C according to the Baranyi (solid lines) and Huang prediction model (dashed lines). The points ◊ represent the observed values of colony diameter of *M. circinelloides* on CBA without NaCl and points Δ represent the observed values of colony diameter of *M. circinelloides* on CBA agar with 1 % NaCl.

The traditional approach in predictive mycology research is to observe fungal growth on a solid agar surface under different selected constant environmental conditions, such as isothermal conditions, to determine the kinetic parameters of a suitable primary model after observations are made. This study aimed to evaluate the growth response of *M. circinelloides* on a cheese-based medium (CBA) at temperatures ranging from 6 to 37 °C, to compare the performance of the Baranyi model (BM) and the Huang model (HM). Representative fits of these primary models to experimental data at 6, 21 and 33 °C are presented in Fig. 1. Similar behaviour was observed for the other conditions tested. The estimated averages of the main parameters (*sgr* and *λ*) from the primary modelling are summarised in Table 1.

**Table 1:** Average surface growth parameters of *M. circinelloides* 1L on CBA.

*T* (°C)	NaCl	*sgr* (mm.h^−1^)		*λ* (h)		*d*_max_ (mm)	*R*^*2*^		*RMSE* (mm)	
		BM	HM	BM	HM		BM	HM	BM	HM
**6**	0	0.080* ±<0.001	0.077 ±<0.001	139.9 ± 3.7	134.1 ± 5.9	113.6 ± 2.0	9.995 × 10^−1^	9.998 × 10^−1^	0.863	1.666
	1	0.064* ±<0.001	0.062 ±<0.001	182.2 ± 13.6	171.5 ± 14.3	104.2 ± 1.3	9.984 × 10^−1^	9.965 × 10^−1^	1.469	2.461
**8**	0	0.117* ±<0.001	0.115 ±<0.001	119.1 ± 2.7	113.0 ± 4.5	122.0 ± 1.8	9.998 × 10^−1^	9.995 × 10^−1^	0.619	0.894
	1	0.078 ± 0.003	0.077 ± 0.003	123.4 ± 5.2	120.0 ± 5.2	93.1 ± 3.2	9.948 × 10^−1^	9.941 × 10^−1^	2.422	2.627
**12**	0	0.162* ±<0.001	0.159 ± 0.001	67.9 ± 4.2	63.5 ± 3.5	121.1 ± 1.6	9.996 × 10^−1^	9.995 × 10^−1^	0.744	0.860
	1	0.090 ± 0.002	0.086 ± 0.002	89.4 ± 11.3	82.6 ± 11.0	69.4 ± 2.2	9.946 × 10^−1^	9.938 × 10^−1^	1.889	2.042
**15**	0	0.183 ± 0.004	0.176 ± 0.004	45.2 ± 2.1	41.2 ± 4.6	113.9 ± 3.6	9.971 × 10^−1^	9.963 × 10^−1^	2.228	2.577
	1	0.097 ± 0.004	0.094 ± 0.004	67.1 ± 4.0	62.2 ± 3.7	81.3 ± 1.6	9.970 × 10^−1^	9.969 × 10^−1^	1.586	1.634
**18**	0	0.207 ± 0.005	0.200 ± 0.006	34.2* ± 1.6	31. 8 ± 0.6	111.6 ± 1.7	9.984 × 10^−1^	9.970 × 10^−1^	1.559	2.200
	1	0.108 ± 0.003	0.105 ± 0.003	62.7 ± 7.2	58.0 ± 5.4	90.6 ± 1.3	9.962 × 10^−1^	9.963 × 10^−1^	2.151	2.143
**21**	0	0.211* ± 0.004	0.198 ± 0.004	7.9 ± 1.3	8. 6 ± 1.0	91.5 ± 0.9	9.933 × 10^−1^	9.903 × 10^−1^	2.850	3.514
	1	0.116 ± 0.004	0.112 ± 0.003	54.5* ± 1.5	50.7 ± 2.0	84.6 ± 1.2	9.979 × 10^−1^	9.981 × 10^−1^	1.506	1.463
**25**	0	0.215 ± 0.002	0.206 ± 0.006	27.3 ± 1.7	18.5 ± 3.8	95.2 ± 1.5	9.931 × 10^−1^	9.879 × 10^−1^	2.902	3.876
	1	0.126 ± 0.006	0.122 ± 0.006	49.9 ± 3.3	44. 7 ± 4.1	81.6 ± 4.0	9.907 × 10^−1^	9.999 × 10^−1^	2.990	3.191
**30**	0	0.289 ± 0.026	0.272 ± 0.024	31.6 ± 3.7	26.0 ± 3.2	103.4 ± 4.0	9.769 × 10^−1^	9.766 × 10^−1^	6.406	6.663
	1	0.142* ± 0.003	0.136 ± 0.002	44.2* ± 0.2	41.0 ± 0.7	56.0 ± 1.9	9.976 × 10^−1^	9.969 × 10^−1^	1.058	1.213
**33**	0	0.276 ± 0.011	0.264 ± 0.011	27.0 ± 3.1	24.1 ± 2.7	92.7 ± 3.3	9.891 × 10^−1^	9.876 × 10^−1^	3.841	4.233
	1	0.162 ± 0.004	0.155 ± 0.005	45.8 ± 4.5	42.8 ± 4.5	52.2 ± 3.2	9.915 × 10^−1^	9.915 × 10^−1^	1.822	1.874
**35**	0	0.258* ± 0.002	0.243 ± 0.003	22.4 ± 2.0	18.2 ± 1.7	93.8 ± 3.2	9.972 × 10^−1^	9.955 × 10^−1^	1.961	2.595
	1	0.048 ± 0.003	0.049 ± 0.003	nl	nl	11.2 ± 0.6	9.158 × 10^−1^	9.247 × 10^−1^	0.575	0.567
**37**	0	0.067 ± 0.006	0.066 ±<0.001	24.5 ± 0.7	24.5 ± 0.9	18.4 ± 2.5	9.365 × 10^−1^	9.380 × 10^−1^	1.548	1.570

*T* – incubation temperature; *sgr* – surface growth rate; *λ* – lag phase duration; nl – no lag; *d*_max_ – maximum diameter of colonies in stationary phase*;* BM – parameters calculated using Baranyi model; HM – parameters calculated using Huang model. Superscript * indicates a statistically significant difference between the parameter fitted with BM and the relevant parameter fitted with HM (*α* = 0.05).

The experimental data on *M. circinelloides* growth showed a typical fungal growth curve, as has been reported by various authors [35,36,[57], [58], [59]]. A lag phase was observed before the formation of a visible colony and was followed by linear growth in all cases. Subsequently, a noticeable deceleration of growth was observed in the stationary phase. However, the stationary phase was not achieved at 8 and 12 °C in a medium without salt addition due to the limited growth surface on Petri dishes of diameter 130 mm used in these trials.

Approximately after 7 days, this dairy isolate of *M. circinelloides* can grow at a rate of 1.8–1.5 mm d^−1^ even at the lowest temperature of 6 °C, which is consistent with Gougouli et al. [58] and Snyder et al. [16], who reported that the growth response of *M. circinelloides* is restricted at temperatures below 6 °C. On the other hand, the result showed that an increase in temperature to 30 °C or 33 °C leads to the fastest growth of the organism at levels of 6.9 mm d^−1^ to 6.3 mm d^−1^. However, a subsequent increase in temperature to 35 °C resulted in a decrease in growth rate. According to the experiments, no growth was observed even after 1 month of incubation at 37 °C on CBA with 1 % NaCl. A similar pattern was reported by Gougouli et al. [58], who did not observe any growth of *M. circinelloides* in yoghurt with a 10 % fat content at 37 °C.

Through the statistical parameters obtained by fitting both models to growth curves, it can be noticed that both models fit well to the experimental data, with *R*^*2*^ values higher than 9.158 × 10^−1^ for Baranyi curves and *R*^*2*^ higher than 9.247 × 10^−1^ for those of the Huang model. The resulting Baranyi curves were almost similar to those of the Huang model with a mean *RMSE* of 1.281 mm (*n* = 83), which was slightly lower than the mean *RMSE* value (1.758 mm; *n* = 83) of the Huang model. However, at some temperatures (at 18, 21 and 35 °C in a CBA with 1 % NaCl), the *RMSE* values of the Baranyi model resulted in relatively higher *RMSE* values compared to those of the Huang model. Several studies have reported similar values of *R*^*2*^ and *RMSE* values for fungal growth and have concluded that the fit quality is appropriate and acceptable [30,44,57,[60], [61], [62], [63]].

In general, the results of this study indicate that slightly higher surface growth rates and lag phases were obtained when the Baranyi model was compared to the Huang model. These findings are in agreement with previous studies [44,[64], [65], [66]]. The statistical analysis of growth kinetics performed using ANOVA revealed that there were no significant differences in the effect of model selection on the mycelial growth rate and lag phase duration at an *α* level of 0.05 in most data sets, as has been reported by various authors [64,[66], [67], [68]]. Overall, the values for surface growth rate and lag phase duration were more influenced by temperature than by model selection. These findings coincide with Juneja et al. [64], who reported that primary growth data were affected by temperature rather than model choice.

The slightly lower values of *R*^*2*^ from Table 1 for the Huang model can be attributed to the transition coefficient *α* that promotes a sharp but smooth, transition from the lag to the exponential phase. On the other hand, Huang's modelling approach has a more clearly identifiable lag phase, which is distinguishable from the exponential phase of growth. Other studies have also confirmed these findings [[67], [68], [69], [70], [71]]. On the other hand, the Baranyi model demonstrated slightly improved performance on the transition between the exponential phase and stationary growth phase. Hong et al. [72] demonstrated a similar pattern of transition from exponential to stationary phase of growth curves for *Clostridium sporogenes* analysed with the Baranyi model and Huang model.

**Fig. 2:**
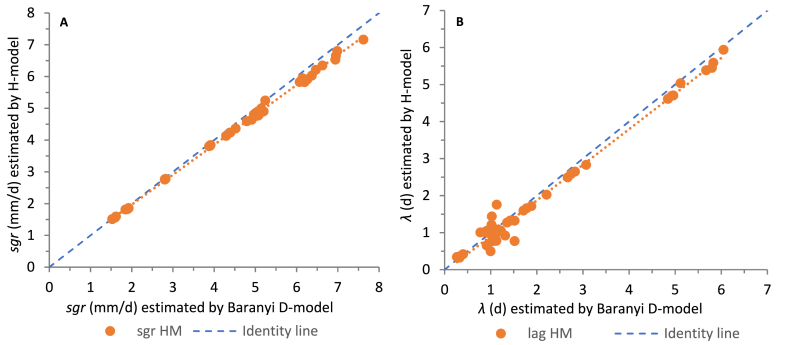
Comparison of the growth parameters as estimated with the Baranyi and Huang primary models.

The comparison between estimated *sgr* and *λ* using the Baranyi and Huang model is shown in Fig. 2A and B, respectively. It seems that both growth parameters estimated using HM are negligibly underestimated as compared with the BM parameters. The coefficients of determination, as well as the standard errors of their linear relations, are close to 1 (*R*^*2*^ = 9.984 × 10^−1^ and 9.823 × 10^−1^; *SE* = 0,07 mm d^−1^ and 5.4 d) for *sgr* and *λ*, respectively.

### 3.2 Secondary modelling of *M. circinelloides* growth

Experimental observations indicate that the incubation temperature influenced *M. circinelloides* growth parameters. In this study, the surface growth rate (*sgr*) and the natural logarithm of the reciprocal lag values (ln 1/*λ*) estimated from the Baranyi and Huang models were fitted using the secondary cardinal model equation (CM; Eqs. (5), (6))).

**Fig. 3:**
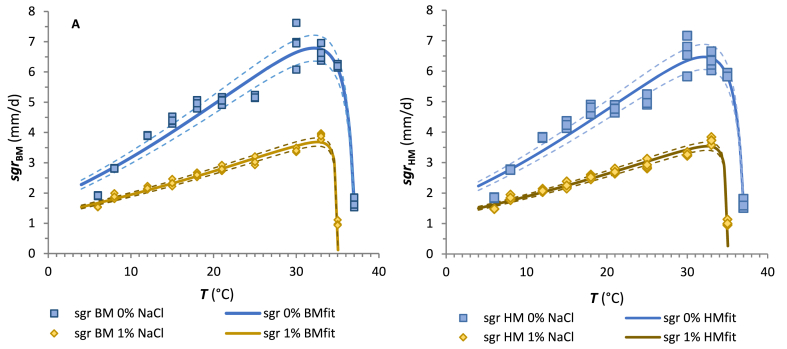
Plots of the surface growth rates of the *M. circinelloides* 1L versus temperature (6–37 °C) on CBA without the addition of NaCl (blue line) and with 1 % addition of NaCl (yellow line) obtained from the Baranyi model (A) and Huang model (B). The symbols ◊ and □ indicate the experimental *sgr* at each incubation temperature. Solid lines represent the fitted model estimates according to the CM. Dashed lines represent predicted sgr values with added or subtracted RMSE values. (For interpretation of the references to colour in this figure legend, the reader is referred to the Web version of this article.)

**Fig. 4:**
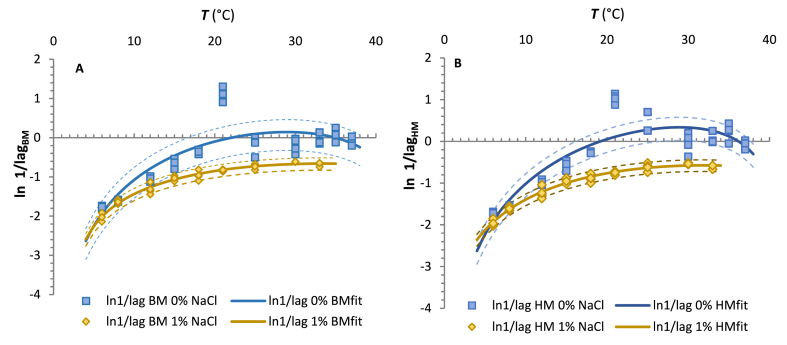
Plots of the natural logarithm of the reciprocal lag phase duration of the *M. circinelloides* 1L versus temperature (6–37 °C) on CBA without the addition of NaCl (blue line) and with 1 % addition of NaCl (yellow line) obtained from Baranyi model (A) and Huang model (B). The symbols ◊ and □ indicate the experimental values at each incubation temperature. Solid lines represent the fitted model estimates according to the CM. Dashed lines represent predicted *sgr* values with added or subtracted RMSE values. (For interpretation of the references to colour in this figure legend, the reader is referred to the Web version of this article.)

The output of surface growth rate and lag phase modelling of *M. circinelloides* growth on CBA for both modelling approaches is presented in Fig. 3, Fig. 4, respectively. As expected, the *sgr* increased with an increase in temperature up to optimum and then decreased beyond the physiological limits of the isolate. The duration of the lag phase showed a reverse pattern of response, the lag times decreased with increasing temperature and slightly increased in the area beyond the optima towards the maximum values of temperature. The values of the CM parameters are summarised in Table 2.

**Table 2:** Mathematical indices and validation factors of the cardinal model (CM) which describe the effects of temperature and NaCl addition on *M. circinelloides* 1L mycelial growth on CBA.

	Baranyi model				Huang model			
Parameter	0 % NaCl		1 % NaCl		0 % NaCl		1 % NaCl	
	CM_*sgr*_	CM_*ln(1/λ)*_	CM_*sgr*_	CM_*ln(1/λ)*_	CM_*sgr*_	CM_*ln(1/λ)*_	CM_*sgr*_	CM_*ln(1/λ)*_
***sgr***_***opt***_ (mm.d^−1^)	6.789 ± 0.117	–	3.686 ± 0.053	–	6.469 ± 0.113	–	3.537 ± 0.050	–
***1/λ***_***opt***_ (1/d)	–	1.192 ± 0.148	–	0.518 ± 0,019	–	1.401 ± 0.149	–	0.564 ± 0.017
***T***_***min***_ (°C)	−28.63 ± 1.24	−0.30 ±0.01	−43.85 ± 2.46	1.86 ±0.36	−29.52 ±1.34	−0.36 ±0.01	−44.60 ± 2.64	−2.17 ±0.06
***T***_***opt***_ (°C)	32.15 ±0.10	28.83 ± 0.32	32.55 ± 0.13	33.22 ± 0.80	32.08 ±0.10	28.90 ±0.27	32.47 ±0.13	31.70 ±0.72
***T***_***max***_ (°C)	37.22 ±0.02	43.47 ± 1.61	35.06 ± 0.01	64.79 ± 3.09	37.23 ±0.02	41.44 ±0.87	35.06 ±0.01	58.38 ±3.70
***R***^***2***^	9.391 × 10^−1^	7.163 × 10^−1^	9.734 × 10^−1^	9.578 × 10^−1^	9.365 × 10^−1^	7.828 × 10^−1^	9.714 × 10^−1^	9.700 × 10^−1^
***RMSE***	0.429	0.426	0.136	0.081	0.413	0.379	0.133	0.078

CM_s*gr*_ – estimated CM parameters and statistical indices for surface growth rate (*sgr*) modelling of *M. circinelloides* against temperature; CM_*ln(1/λ)*_ - estimated CM parameters and statistical indices for lag time (*λ*) modelling; *sgr*_*opt*_–surface growth rate at the temperature optimum; *T*_min_ – minimum temperature (theoretical value); *T*_*opt*_ – optimal temperature; *T*_max_ – maximum temperature*; R*^*2*^ – coefficient of determination; *RMSE* – root mean square error.

Among them, the *T*_min_ values estimated by the CM rate model appear unrealistically negative, as well as lower values at higher NaCl concentrations. However, it is generally known that the *T*_min_ provided by the CM model is considered theoretical. For example, for pseudomonades, Tarlak [73] also estimated low *T*_min_ values of −12.6 to −17.9 °C.

### 3.3 Time to create visible colonies

**Fig. 5:**
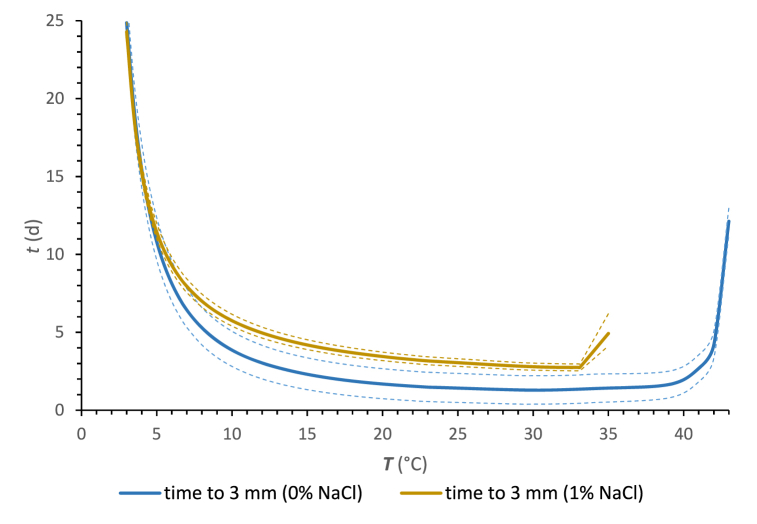
The prediction of the time required for *M. circinelloides* 1L to yield visible (3 mm) colonies on the CBA depending on temperature and NaCl addition. Solid lines represent the predicted *t*_3_ and dashed lines represent predicted *t*_3_ values with added or subtracted RMSE values.

The cardinal parameters for the lag phase and surface growth rate were used to predict the times required for visible colonies that can be useful, e.g., in dairy practice. Generally, such predictions are important for fast-growing fungal species such as *M. circinelloides* representing lower fungi [6]. Referring to microscopic fungi, the 3 mm colony is considered visible (*t*_3_) under a set of specific environmental conditions [24,58,61]. For this purpose, the time predictions with temperature are shown in Fig. 5. Based on the estimated results, the *t*_3_ is minimized when *M. circinelloides* displays higher surface growth rates. Other studies have also confirmed these findings [58,61].

The growth data presented in this work confirmed the rapid growth characteristic of *M. circinelloides* is characterised by a short time to yield a visible colony. *M. circinelloides* exhibits a faster growth rate compared to *G. candidum*, a mould commonly referred to as the "machinery mould" [74]. Koňuchová and Valík [59] reported that the calculated *t*_3_ values for two strains of *G. candidum* on skim milk agar at *a*_*w*_ 0.97 at 10 °C were in the range of 8.6–12.1 d. In contrast, *M. circinelloides* needed only 4 d to create a visible colony in CBA at the same *a*_*w*_ and temperature. Burgain et al. [75] reported calculated *t*_3_ values of 2.3 days for *Penicillium chrysogenum* 738 on potato dextrose agar at *a*_*w*_ 0.970 at 25 °C. Conversely, *M. circinelloides* studied in the present study required only 1.3 d to create a visible colony in CBA under analogous *a*_*w*_ and temperature conditions. These observations align with those of Gougouli et al. [58], underlining the classification of *M. circinelloides* among dairy spoilage fungi characterized by the shortest *t*_3_ values. From the practical point of view and taking the sensitivity of this mould to NaCl, e.g. almost 50 % reduction of *sgr*_opt_ caused by a concentration of 1 %, the above data support the decision on the frequency of washing the surface of cheese lumps with a salt solution.

### 3.4 Validation at the dynamic temperature change

**Fig. 6:**
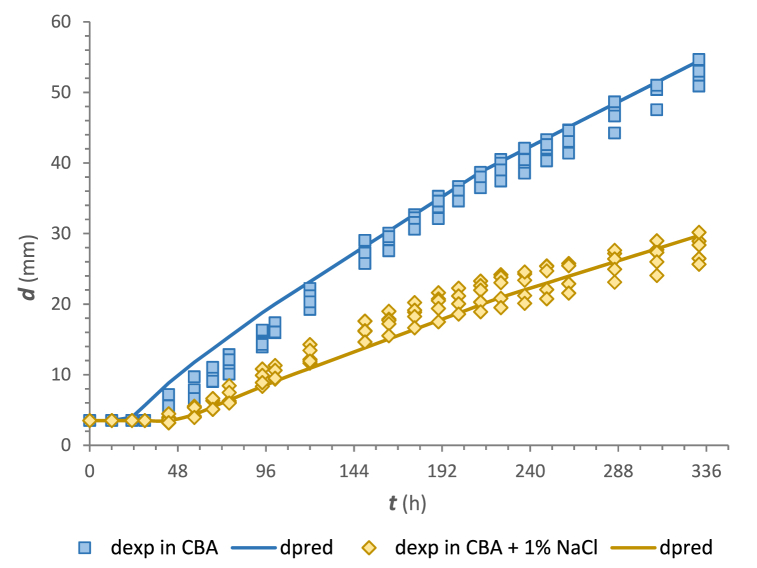
Validation of the CTMI growth models for *M. circinelloides* on CBA at dynamic temperature changes (25 °C/48 h; 18 °C/48 h; 15/120 h; 10 °C/120 h).

For validation, additional experiments with programmed temperature changes were performed under conditions of 0 and 1 % NaCl on CBA. The combination of time and temperature (25 °C and 18 °C, each for 2 d; 15 °C and 10 °C, each for 5 d) reflected the fermentation and ripening conditions for the artisan production of ewe lump cheese, which is produced from raw and pasteurised milk in Slovakia. The results of validation using CMs with the parameters from Table 2, including lag models, are shown in Fig. 6 and the validation indices are summarised in Table 3.

**Table 3:** Mathematical indices and validation factors of cardinal model (CM) which describe the effects of temperature and NaCl addition on *M. circinelloides* 1L mycelial growth on CBA at dynamic temperature changes.

	0 % NaCl	1 % NaCl
***R***^***2***^	9.917 × 10^−1^	9.660 × 10^−1^
***RMSE***	2.349	1.772
***B***_***f***_	1.132	0.950
***A***_***f***_	1.221	1.119
***%D***_***f***_	−22.058	−11.947

*R*^*2*^ – coefficient of determination; *RMSE* – root mean square error; *A*_*f*_ – accuracy factor; *B*_*f*_ – bias factor; *%D*_*f*_ – % discrepancy.

The *RMSE* values for growth on CBA without NaCl and with 1 % NaCl addition were 2.349 and 1.772 mm, respectively. Both the graphical evaluation and satisfactory *B*_*f*_ and *A*_*f*_ limits demonstrated an acceptable model performance [76,77] in predicting the growth of *M. circinelloides* on CBA. Meanwhile, the calculated *B*_*f*_ value for the growth of *M. circinelloides* on CBA without NaCl addition was 1.132. Thus, the observed colony diameters are overestimated by less than 13.2 % and their calculation based on secondary CM models would lead to a “fail-safe” prediction. In the case of the nonisothermal growth on CBA with 1 % NaCl, the calculated *B*_*f*_ value indicated that the model slightly underestimates observed values and would provide a “fail-dangerous” prediction. In a modelling study carried out on apple puree agar medium, using the Ratkowsky model comparison of predicted and estimated growth rates of *Penicillium expansum* resulted in *B*_*f*_ between 0.91 and 1.14 [78]. The *B*_*f*_ values obtained in our study are of the same magnitude, if not closer to 1 as compared to the previously reported study. The *A*_*f*_ factors of 1.221 and 1.119 showed that predicted values for the growth of *M. circinelloides* on CBA were a maximum of 22.1 % different (either smaller or larger) from observed values. These *A*_*f*_ values are in close agreement with those obtained by Judet-Correia et al. [79], who reported *A*_*f*_ of 1.11 and 1.29 for the growth rate estimates of *Penicillium expansum* and *Botrytis cinerea*.

## 4 Conclusions

Overall, the present study has successfully demonstrated the effective application of two primary models (Baranyi and Huang) to predict the growth of *M. circinelloides* on the surface of a cheese-based medium under isothermal conditions and two different levels of *a*_*w*_. The application of the Huang model to the mycelial growth of this cheese-associated fungus is an element of the originality of the present approach in the context of predictive mycology. In terms of prediction parameters, Baranyi and Huang models showed a similar degree of prediction capacity in predicting fungal growth. The maximum surface growth rate was then fitted to the secondary cardinal model to describe the colony growth rate as a function of temperature and *a*_*w*_. Based on modelling analysis, we concluded that the most suitable growing conditions were 0.974 *a*_*w*_ (unmodified growth medium) and the temperature around 32 °C, resulting in the highest growth rate. The cardinal model was able to describe the impact of temperature and *a*_*w*_ within the tested ranges on *sgr* with *R*^*2*^ values higher than or equal to 9.365 × 10^−1^ and *RMSE* lower than or equal to 0.429 mm d^−1^. Validation studies under dynamic temperature conditions indicated that the model used satisfactorily predicted the surface growth behaviour of *M. circinelloides*. The statistical indices obtained for the growth validation were within the range of 0.950 < *B*_*f*_ < 1.132 and 1.119 < *A*_*f*_ < 1.221. The limitation of the study lies in the use of an artificial growth medium that has not included interactions with a background cheese microbiota. However, this fact can be considered a “fail-safe”, which is still a better case for predictions.

The predictive models used in this study have the potential to be used as a simulation tool for dairy processors, enabling them to monitor the microbiological quality of cheese before its distribution to consumers. The growth prediction data of the lower fungus studied, mainly the times required to create visible colonies, can also help cheese producers decide on the frequency of the washing step of cheese surfaces with salt solution, which is a normal part of the production process in the early stage of ripening. It is enabled because of the sensitivity of *M. circinelloides* and other frequent fresh cheese contaminants, e.g. *G. candidum,* to NaCl. The results of fast-growing microscopic moulds represented by *M. circinelloides* can be considered to support the development of cheese products, exposure assessment studies, or the formulation of modifications for high-quality cheese products. However, these ideas can become real if the fundamental role of prerequisites such as GMP and GHP is applied effectively.

## Funding sources

The research was funded by the APVV-190031 and 10.13039/501100006109VEGA 1/0132/23.

## Data availability

The data that support the findings of this study are available from the corresponding authors, [MK, ĽV], upon reasonable request.

## CRediT authorship contribution statement

**Martina Koňuchová:** Writing – original draft, Supervision, Methodology, Investigation, Formal analysis, Conceptualization. **Agáta Boháčiková:** Investigation, Formal analysis, Data curation. **Ľubomír Valík:** Writing – review & editing, Visualization, Validation, Supervision, Software, Project administration, Methodology, Funding acquisition, Conceptualization.

## Declaration of competing interest

The authors declare that they have no known competing financial interests or personal relationships that could have appeared to influence the work reported in this paper.
